# One-Month Follow-Up of Patients with Unspecified Abdominal Pain Referring to the Emergency Department; a Cohort Study

**Published:** 2019-08-17

**Authors:** Seyed Mohammad Hoseininejad, Reza Jahed, Mohammad Sazgar, Fatemeh Jahanian, Seyed Jaber Mousavi, Syed Hosein Montazer, Touraj Asadai, Hamed Aminiahidashti

**Affiliations:** 1 **Gut and Liver Research Center, Mazandaran University of Medical Sciences, Sari, Iran.**; 2 **Student Research Committee, Mazandaran University of Medical Sciences, Sari, Iran.**; 3 Emergency Department, Mazandaran University of Medical Sciences, Sari, Iran.; 4 **Department of Community Medicine, Mazandaran University of Medical Sciences, Sari, Iran.**

**Keywords:** Abdominal pain, patient discharge, follow-up studies, emergency service, hospital

## Abstract

**Introduction:**

About one third of patients referring to emergency department (ED) with abdominal pain, are discharged without a definite diagnosis. This study aimed to investigate the one-month outcome of patients with unspecified abdominal pain.

**Methods:**

This cohort study was conducted on subjects who were evaluated in ED with unspecified abdominal pain and were referred to the gastroenterology clinic and followed for one month. Finally, they were divided into two groups of cases with clear cause of abdominal pain and unclear cause of abdominal pain and patients’ characteristics were compared between the groups.

**Results:**

150 cases with the mean age of 40.68 ± 18.34 years were studied (53.3% female). After one month, 67 (44.7%) patients still complained of abdominal pain. A definitive cause of abdominal pain was established in 88 (58.7%) cases. There was not any significant difference between groups regarding, sex distribution (p = 012), duration of pain (p = 0.11), history of previous similar pain (p = 0.136), pain radiation (p = 0.737), length of hospital stay (p = 0.51), and presence of anorexia (p = 0.09), nausea and vomiting (p= 0.50), fever (p = 1.0), diarrhea (p = 0.23), and constipation (p = 0.07). There was a significant difference between the groups regarding location of pain (p = 0.017), age (p = 0.001) and history of comorbid diseases (p = 0.046). The predictive factors of finding a clear cause for abdominal pain in one-month follow-up, were leukocytosis (OR: 5.92 (95% CI: 2.62 – 13.39); p < 0.001), age (OR: 2.78 (95% CI: 1.15 – 6.71); p = 0.023), and outpatient follow-up (OR: 1.04 (95% CI: 1.02 – 1.07); p < 0.001).

**Conclusion:**

Approximately, 40% of patients who were discharged with unspecified abdominal pain did not receive a clear diagnosis after one month of follow-up. Older age, leucocytosis in initial evaluations, and outpatient follow-up increased the probability of finding a clear cause for abdominal pain in the mentioned cases.

## Introduction

Acute abdominal pain is defined as a non-traumatic pain that has begun less than 5 days before (1). It is one of the most common clinical complaints of patients that refer to the emergency department (ED) and is the cause of about 7-10% of all ED referrals (2, 3). Despite the high frequency of these referrals to the ED, there is no definitive diagnostic way to distinguish between emergency and non-emergency causes for abdominal pain (1). 28% to 36% of these patients are discharged without definite diagnosis (4-6). The unclear cause of abdominal pain in the ED is commonly associated with insufficient history taking, inadequate use of diagnostic tests, and problems related to follow-up visit and test results (7). This challenge in differential diagnosis of abdominal pain can result in adverse consequences and legal and medical lawsuits (8). Although thorough physical examination, careful observation, and repeated diagnostic tests are effective ways to reduce the risk of harmful and unintended consequences (9), in many cases, patients are dissatisfied with the long waiting time in ED (10). In addition, there is no specific guideline for dealing with patients with abdominal pain without definite diagnosis in ED (11). Based on above-mentioned points, this study aimed to investigate the one-month outcome of patients with abdominal pain without definite diagnosis in ED. 

## Methods

***Study design and setting***

This cohort study was conducted in Gastroenterology Research Center, Imam Khomeini Hospital, Mazandaran University of Medical Sciences, Sari, Iran from March 2016 to February 2017. Imam Khomeini Educational Hospital is a public and tertiary referral center in Mazandaran province with 400 beds, and the Gastroenterology Center is one of the first research centers in this area. This study was conducted on subjects who were admitted to the ED with abdominal pain with ICD 10 code R10.4. All patients with abdominal pain (ICD10 code R10) were visited by an emergency medicine specialist and they were admitted to the observation unit of ED for at least 6 hours and the serious causes of abdominal pain were ruled out. Patients with abdominal pain who were diagnosed at this stage were excluded. Patients with unclear diagnosis were referred to the gastroenterology clinic and followed for one month. Finally, patients were divided into two groups of patients with clear cause of abdominal pain and unclear cause of abdominal pain after one-month follow-up and patients’ characteristics were compared between groups.

All information of the participants was confidential and they were enrolled into the study after obtaining the consent of the patient or their relatives. This study was approved by the Ethics Committee of Mazandaran University of Medical Sciences with the reference code: IR.MAZUMS.REC.95-1625.

***Participants***

All patients with non-traumatic abdominal pain who were discharged without a definite diagnosis despite physical examination, laboratory, and imaging studies (ICD 10 code R10.4) were included in the study. Addicts or those with a history of addiction, pregnant women, patients with abdominal pain following trauma, those who left the ED against medical advice, and those who were lost to follow-up after a month, were excluded. In addition, patients who were under observation for 6 hours and a definite cause was diagnosed and those whose clinical condition was not suitable for discharge and were admitted to ward according to the decision of the emergency medicine specialist were excluded. 

***Patients’ Follow-up***

A summary of the patient's ED clinical profile was given to each patient and referred to the gastroenterology clinic. Every week the patients’ data were collected from the gastroenterology clinic and they were contacted via phone after a month. In addition, a visit was arranged with the patient or their relatives, and the completed investigation, definitive diagnosis, recovery, mortality and morbidity were questioned and recorded. 

***Data Gathering***

Definitive diagnosis, age, sex, duration of pain, severity and location of pain, duration of admission, underlying disease, accompanying signs and symptoms, laboratory findings, as well as the results of one-month follow-up regarding readmission, pain status, cause of pain, and mortality were collected using a predesigned checklist. Data from each of the groups were collected by the main author without any intervention in the patient's treatment and care process.

**Statistical Analysis**

All data were collected and recorded in SPSS statistical software version 22.0. Quantitative data were described as mean ± standard deviation. Frequency and percentage of variables were used to describe qualitative data. Chi square (X^2^) test and logistic regression analysis were used to determine the associated factors of the probability to reach a definite diagnosis. P value less than 0.05 was considered statistically significant.

## Results

***Baseline characteristics of studied patients***

**Figure 1:**
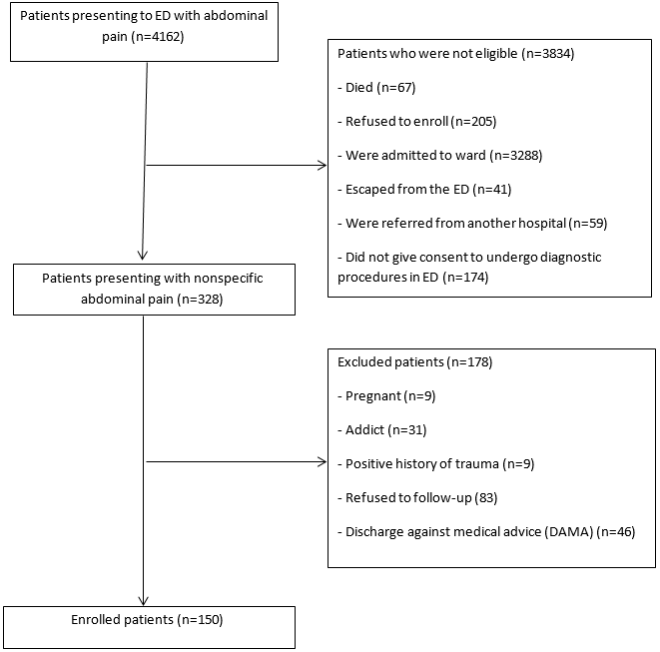
Patient selection flowchart

39,817 patients were admitted to the emergency department during the study period (4162 cases was classified as ICD10 code R10). Only 328 (7.77%) patients were eligible for enrollment to the study (ICD10 code R10.4) and, finally, a study was conducted on 150 patients (Figure 1). The subjects’ mean age was 40.68 ± 18.34 (6-85) years (53.3% female). In a re-visit to gastroenterology clinic after one month, 83 (55.3%) patients noted their pain was relieved, and 67 (44.7%) patients still complained of their pain. Meanwhile, 63 (42%) patients complained of multiple referrals due to abdominal pain. A definitive cause of abdominal pain was established in 88 (58.7%) cases, yet the cause of abdominal pain was still unclear in 62 (41.3%) patients. Most patients were diagnosed with renal colic (16.6%), followed by biliary colic (8.7%) and ovarian cyst (5.3%). 

**Table 1:** Comparing the baseline characteristics of patients with clear and unclear cause of abdominal pain

**Variable**	**Cause of abdominal pain**		**P value**
	**Clear (n = 88)**	**Unclear (n = 62)**	
**Age (years)**			
Mean ± SD	44.9 (18.7)	34.5 (15.9)	0.001
**Gender**			
Male	45 (51.1)	25 (40.3)	0.12
Female	43(48.9)	37(59.7)	
**Duration of pain before admission (hours)**			
Mean ± SD	35.1(47.5)	58.7(128.1)	0.11
**History of similar pain**			
Yes	17 (70.8)	7 (29.2)	0.136
No	71 (56.3)	55 (43.7)	
**Length of hospital stay (days)**			
Mean ± SD	6.7 (4.4)	6.1 (5.1)	0.51
**History of comorbidity** ^1^			
Yes	48 (54.5)	23 (37.1)	0.046
No	40 (50.6)	39 (49.4)	
**Pain radiation**			
Yes	6 (66.7)	3 (33.3)	0.737
No	82 (58.2)	59 (41.8)	
**Presenting sign and symptom**			
Anorexia	13 (14.8)	16 (25.8)	0.09
Nausea and vomiting	51(58.0)	32 (51.6)	0.50
Fever	7 (8.0)	5 (8.1)	1.0
Diarrhea	5 (5.7)	7 (11.3)	0.23
Constipation	5 (5.7)	9 (14.5)	0.08
**Laboratory findings**			
Leukocytosis^2^	33 (40.7)	15 (25.9)	0.07
Anemia^3^	32 (39.5)	25 (43.1)	0.32

Data are presented as mean ± standard deviation (SD) or number (%). 1: diabetes mellitus, hypertension, renal stone, menorrhagia, ovarian cyst, discopathy, GI bleeding, end stage renal disease, and etc., 2: White blood cell count ≥1110^3^/µL, 3: Hemoglobin˂10 mg/dl.

**Figure 2:**
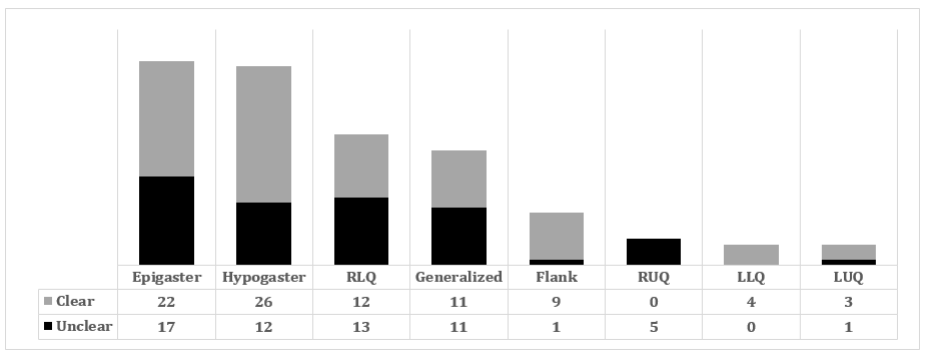
Location of pain in cases with clear and unclear cause of abdominal pain (p = 0.017). RLQ: right lower quadrant of abdomen, LLQ: left lower quadrant of abdomen, RUQ: right upper quadrant of abdomen, LUQ: left upper quadrant of abdomen

**Table 2:** Comparing the one-month outcomes of cases with clear and unclear cause of abdominal pain

Variables	Cause of abdominal pain		P value
	Clear (n = 88)	Unclear (n = 62)	
Pain condition			
**Painless**	55 (62.5)	48 (77.4)	0.07
**Painful**	33 (37.5)	14 (22.6)	
Readmission			
**Yes **	27 (35.5)	40 (64.5)	< 0.001
**No**	61 (69.3)	22 (35.5)	
Mortality			
**Yes**	7 (8.0)	2 (3.2)	0.308
**No**	81 (42.6)	60 (57.4)	

***Comparing the groups***

Table 1 and figure 2 compare the baseline characteristics of patients with clear and unclear causes of abdominal pain. There was not any significant difference between patients with clear and unclear cause of abdominal pain regarding, sex distribution (p = 012), duration of pain ( p = 0.11), history of previous similar pain (p = 0.136), pain radiation (p = 0.737), length of hospital stay (p = 0.51), and presence of anorexia ( p = 0.09), nausea and vomiting (p= 0.50), fever (p = 1.0), diarrhea (p = 0.23), and constipation (p = 0.07).

Patients with clear cause of abdominal pain were older than those with unclear cause (p = 0.001). There was a significant difference between groups regarding the location of pain at the time of presenting to ED (p = 0.017) and history of comorbid disease (p = 0.046). Although the frequency of leukocytosis (WBC > 11,000) was not significantly different between the two groups, mean WBC count on the first day of admission was significantly higher in patients with clear cause (10.5 ± 3.40 versus 9.4 ± 3.17 10^3^/µL; p = 0.043).

Table 2 compares the one-month outcomes of cases with clear and unclear cause of abdominal pain. Based on logistic regression analysis, the predictive factors of finding a clear cause for abdominal pain in one-month follow-up were leukocytosis (OR: 5.92 (95% CI: 2.62 – 13.39); p < 0.001), age (OR: 2.78 (95% CI: 1.15 – 6.71); p = 0.023), and outpatient follow-up (OR: 1.04 (95% CI: 1.02 – 1.07); p < 0.001).

## Discussion

In this study, it was found that approximately 40% of patients who were discharged with abdominal pain without a definite diagnosis did not receive a final definitive diagnosis or clear cause. The most common definite diagnosis in this study was kidney stones. Older age, leucocytosis in initial evaluations, and outpatient follow-up increased the probability of finding a clear cause for abdominal pain in the mentioned patients. 

In some studies, appendicitis (12) and gastric ulcers (13) were reported as the most common causes of abdominal pain without definite diagnosis. In our study, the pain associated with renal stones (renal colic) was the most common cause of abdominal pain, which was similar to the findings of Cervellin et al. (6). It was previously shown that older age is associated with increased hospitalization length and definitive diagnosis (14), which is consistent with the results of our study. In addition, increased likelihood of problems such as mesenteric ischemia, and rupture of aortic aneurysm in cases with underlying diseases such as diabetes and hypertension, makes further workups necessary in these groups of subjects (15, 16). Although in some studies there was no significant relationship between leukocyte count and definitive diagnosis of abdominal pain (13), in our study the increase in white blood cells had a significant relationship with finding a definitive cause for abdominal pain. In the study of Cervellin, 6.9% of patients with abdominal pain returned to the emergency department within the first 5 days, and 7.6% of the patients returned within 5 days to one month after discharge. In the second visit, the initial diagnosis was changed to renal colic in many of these patients (6). 

In the present study 150 patients were discharged, 87 of whom were readmitted to emergency department and 67(44.67%) had definite diagnosis in readmission. In a study, it was shown that pain in the right lower quadrant of the abdomen is likely associated with definitive diagnosis and led to surgery, especially in older people (17). 

Patients with abdominal pain who are discharged without a definite diagnosis are recommended to refer to a medical center if abdominal pain continues for 2 consecutive days (18). In our study, abdominal pain patients discharged without a definite diagnosis who were re-visited in the gastroenterology clinic were more likely to receive a definite diagnosis. This demonstrates the importance of follow-up. If there is suspicion of patients’ inability to follow up, they should remain in the ED under observation and the emergency department should provide the needed facilities. 

Patients with abdominal pain discharged without a definite diagnosis should be carefully evaluated. Patients with older age, readmission, and leukocytosis may be at risk for presence of a clear cause for abdominal pain. So they may benefit from longer observation and consultation for hospitalization. In case of discharge, patients with abdominal pain who are discharged without a definite diagnosis should be monitored by family physicians and/or as an outpatient referring again after a specific interval with specific instructions at the time of discharge. 

## Conclusion:

Approximately 40% of patients who were discharged with abdominal pain without definite cause did not receive a clear diagnosis. Older age, leucocytosis in initial evaluations, and outpatient follow-up increased the probability of finding a clear cause for abdominal pain in the mentioned patients.

## Limitation

There were several limitations in this study. Many of the patients who enrolled did not fully cooperate or provided incorrect information on their diagnostic and therapeutic procedures. Patients' medical records were incomplete in some cases, which caused a limitation in the number of samples.
